# Standardizing HER2 immunohistochemistry assessment: calibration of color and intensity variation in whole slide imaging caused by staining and scanning

**DOI:** 10.1186/s42649-023-00091-8

**Published:** 2023-09-14

**Authors:** Chie Ohnishi, Takashi Ohnishi, Peter Ntiamoah, Dara S. Ross, Masahiro Yamaguchi, Yukako Yagi

**Affiliations:** 1 School of Engineering, Tokyo Institute of Technology, Kanagawa, 226-8503 Japan; 2 Department of Pathology and Laboratory Medicine, Memorial Sloan Kettering Cancer Center, New York, 10065 USA

**Keywords:** Immunohistochemistry (IHC), Whole slide image (WSI), Color and stain intensity calibration, Breast cancer

## Abstract

In the evaluation of human epidermal growth factor receptor 2 (HER2) immunohistochemistry (IHC) — one of the standard biomarkers for breast cancer— visual assessment is laborious and subjective. Image analysis using whole slide image (WSI) could produce more consistent results; however, color variability in WSIs due to the choice of stain and scanning processes may impact image analysis. We therefore developed a calibration protocol to diminish the staining and scanning variations of WSI using two calibrator slides. The IHC calibrator slide (IHC-CS) contains peptide-coated microbeads with different concentrations. The color distribution obtained from the WSI of stained IHC-CS reflects the staining process and scanner characteristics. A color chart slide (CCS) is also useful for calibrating the color variation due to the scanner. The results of the automated HER2 assessment were compared to confirm the effectiveness of two calibration slides. The IHC-CS and HER2 breast cancer cases were stained on different days. All stained slides and CCS were digitized by two different WSI scanners. Results revealed 100% concordance between automated evaluation and the pathologist’s assessment with both the scanner and staining calibration. The proposed method may enable consistent evaluation of HER2.

## Introduction

Whole Slide Imaging (WSI) is a technique to digitalize a glass slide to view a high-resolution digital image. WSI has been implemented into clinical practice, education, and research, and is a promising technology, especially in combination with automated image analysis, as it improves efficiency and may help achieve consistent interpretation. The H&E stained images—the most widely used in clinical assessment—have become the subject of intense research. WSI on H&E stained images is now being used for primary diagnosis. There is an ever-increasing demand for expanding the use of WSI to other types of stained images such as immunohistochemistry (IHC), which is used for the visualization of protein expression. However, the color variability should be addressed before WSI can be reliably used for automated image analysis for IHC.

Color variation of immunohistochemical staining in the tissue specimens is one of the important aspects of pathological assessment. Even US Food and Drug Administration (FDA) cleared or approved workflows may lead to color and intensity differences between stain batches or institutions. Variability in histochemical staining is known to affect the accuracy and reproducibility in clinical practice and research (Gray et al. 2015; Bogen 2019). Slight differences in staining intensity can significantly affect the interpretation of IHC slides, particularly with human epidermal growth factor receptor 2 (HER2) tests that assess cell membranous staining. In digital pathology, digitization of the slides using WSI scanners introduces a further color variation in the scanned image (Gray et al. 2015; Yagi 2011). Such variations can lead to different evaluations by pathologists and image analysis. FDA-approved products have been released for clinical image analysis (Cornish 2020); however, these products are designed for use with specific antibodies/probes or WSI scanners to minimize the impact of image variability, which may limit their use.

We previously proposed a protocol to address the color variation in IHC due to the staining procedure (Ohnishi et al. 2022, 2023). The previous experiment was conducted on HER2 IHC stained tissues. In HER2 IHC, 3,3′-Diaminobenzidine (DAB) stains the membrane region brown, and hematoxylin stains the nuclear region blue or purple for tissue counterstaining. HER2 tests assess the intensity and percentage of membrane staining. For accurate image analysis, calibration of DAB color and intensity is needed. The proposed protocol uses IHC calibrator slides (IHC-CSs) for HER2 made of microbeads coated with different amounts of peptide concentration (Sompuram et al. 2015) to calibrate the DAB color and intensity. In the previous experiment, a single scanner was used to focus on the variation in the staining process, and the effect of device-dependent variation was not examined. Since the protocol uses DAB colors obtained from scanned images, device-dependent color variation may also be calibrated with IHC-CS. Another approach for the color calibration of device-dependent variation, the methods using a color chart slide (CCS) have been reported (Bautista et al. 2014; Clarke et al. 2018).

Here, we describe the application of this protocol to the images scanned with multiple WSI scanners to address the device-dependent color variation. In addition, a comparative experiment was conducted to see if there is any benefit to applying color calibration using CCS. This is the first report showing that the calibration protocol using IHC-CS improved the reliability of the automated HER2 IHC assessment by eliminating the device-dependent variations.

## Materials and methods

### Tissue samples and calibration slides

All slides used in this study were deidentified and breast cancer was targeted. Per the American Society of Clinical Oncology (ASCO) /College of American Pathologists (CAP) HER2 testing guideline (Wolff et al. 2018), all invasive breast cancers are tested for HER2 and semi-quantitatively classified to HER2 status (score 0, 1 + , 2 + , or 3 +). Four cases of breast cancer excisions with HER2 scores of 0, 1 + , 2 + , and 3 + were selected by a senior pathologist. Formalin-fixed paraffin-embedded tissue samples were sectioned at 4 μm.

**Fig. 1:**
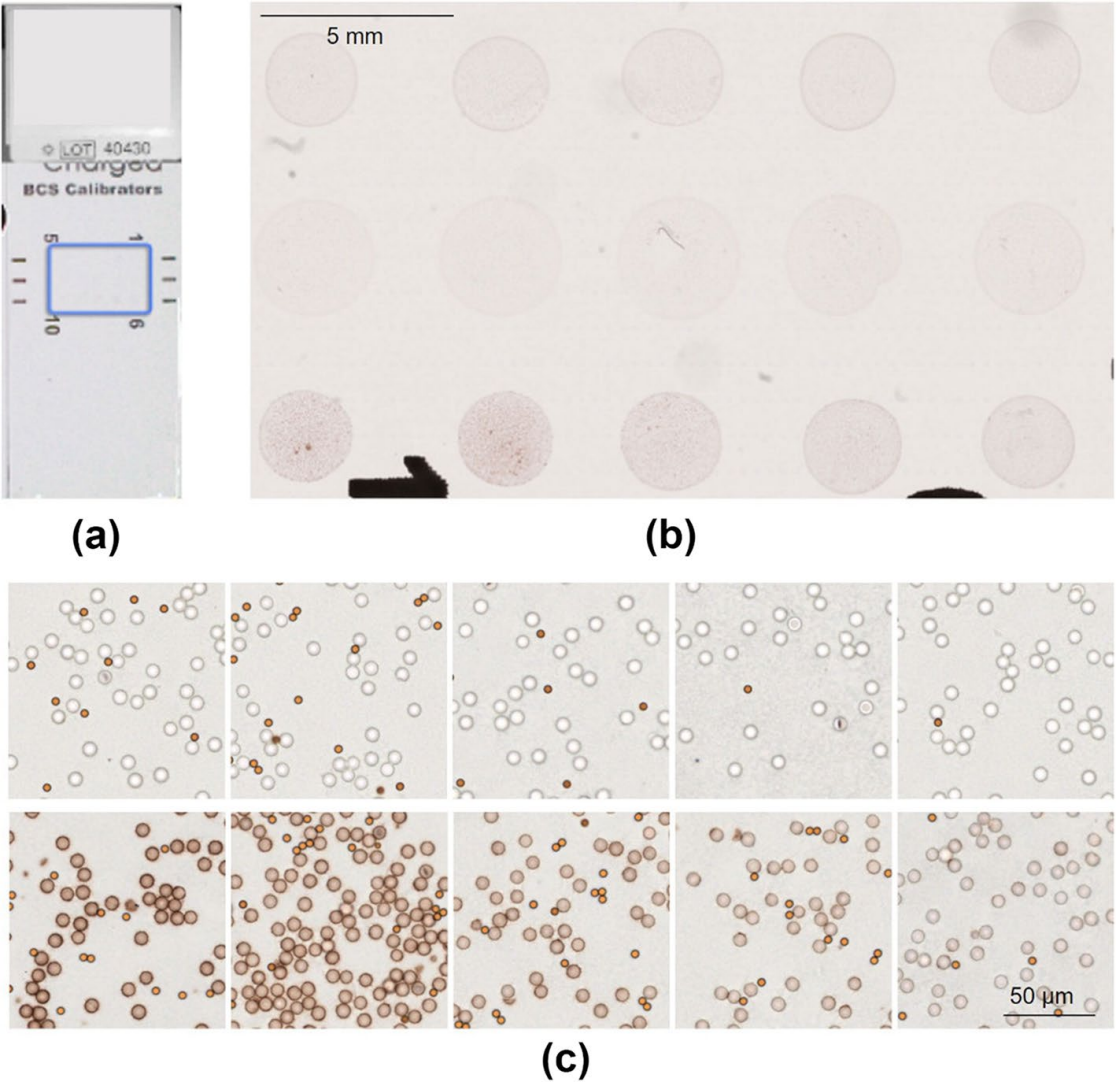
IHC Calibrator Slide for HER2. **a** Slide overview; **b** Whole slide image scanned at a resolution of 0.23 μm/pixel; **c** Microbeads on level 1 to 5 (top right to left), 6 to 10 (bottom right to left)

**Fig. 2:**
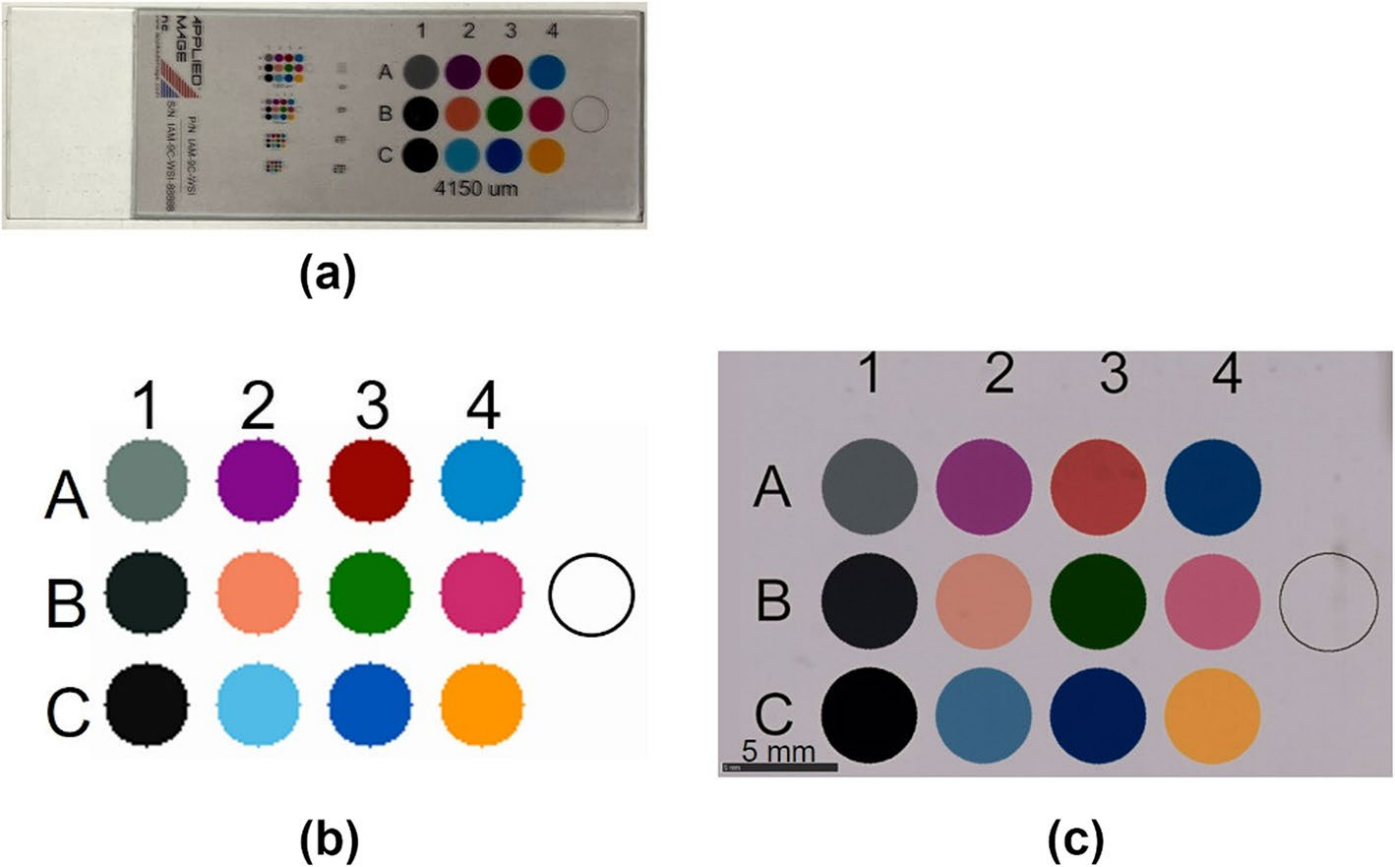
Color Chart Slide. **a** Slide overview; **b** Reference colors calculated from spectral transmission data for each color on the slide, the CIE color-matching function, and spectral distribution of illuminant D65; **c** Whole slide image scanned at a resolution of 0.23 μm/pixel

In this research, two types of calibration slides were used;

- 1. IHC-CS
  The IHC-CS for HER2 (IHC Calibrator, Boston Cell Standards, Massachusetts, USA) was originally designed to maintain reproducible laboratory testing for quality assurance (Sompuram et al. 2015). It comprises two different microbeads (Fig. 1). Larger microbeads are test microbeads coated with 10 different levels of peptide concentration and stained with DAB by IHC staining. Smaller microbeads are colored brown, showing the standard DAB color. The larger microbeads were used in our protocol.
- 2. CCS
  The CCS (IAM-9C-WSI, Applied Image Inc., New York, USA) is designed based on the report (Bautista et al. 2014) to calibrate, standardize, and trace color settings for imaging analysis (Fig. 2). It is made with a typical glass slide embedded with 12 color patches and a background area.

Five slides comprising HER2 0–3 + cases and the IHC-CS were regarded as a dataset. To obtain the slides reflecting the daily variation in staining, the datasets were stained with PATHWAY anti-HER2/neu Antibody (Ventana Medical Systems, Inc., Arizona, USA) on different days. Six datasets were prepared. One case was stained with H&E for evaluation of the CCS. All stained slides and the CCS were digitized by two WSI scanners, Nano Zoomer S60 (Hamamatsu photonics K.K., Shizuoka, Japan) at a resolution of 0.23 μm/pixel and PANNORAMIC 250 Flash III (3DHISTECH Ltd., Budapest, Hungary) at a resolution of 0.18 μm/pixel. The tissue, color chart, and microbead areas were exported from the acquired WSI for image analysis.

### Overview of the proposed calibration protocol

**Fig. 3:**
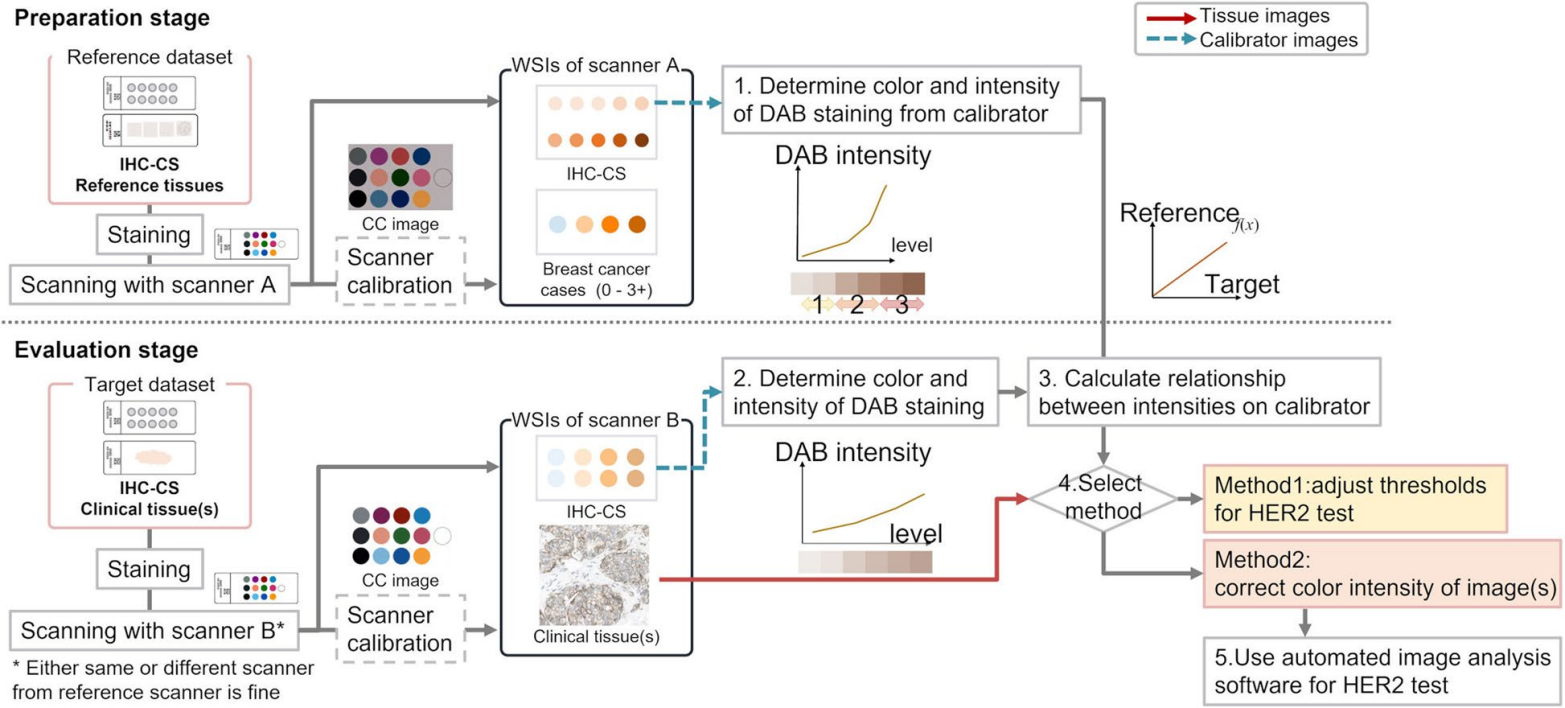
Overview of proposed color and intensity standardization protocol composed of scanner and staining calibration. First, color calibration of the WSI scanner with a color chart slide is performed, then color intensity calibration for staining variation is performed using the IHC calibrator slide. DAB color intensities are obtained from IHC calibrator images (step1–2) and used to calibrate color intensity of tissue images. Method1 is adjusting the thresholds for classifying DAB membranous intensities; Method2 is correcting the color intensity of the image

Using an IHC-CS for automated HER2 assessment, we previously proposed a method of calibrating color variation caused by the staining process (Ohnishi et al. 2022, 2023). Since the DAB color intensities of microbeads in the IHC-CS image correlate with those of the tissue sample images, the DAB color is obtained from the color of microbeads, and the intensity characteristics are derived from the intensities of the different levels of microbeads in the proposed protocol (Fig. 3 steps1–2). Because the IHC-CS was originally designed for the QA/QC of staining, the HER2 status cannot be determined directly from the IHC-CS image. Therefore, the reference DAB-stained tissue data are prepared along with the IHC-CS in advance, and the threshold values for the DAB intensity are determined automatically. Color intensities obtained from the IHC-CS images are used to calibrate the threshold values for HER2 score classification or the tissue images before the automated HER2 score calculation. We had confirmed that the proposed protocol calibrates color and intensity and classifies HER2 status with less variability between datasets.

In the previous experiment, the images used for the HER2 assessment were digitized by the same WSI scanner as the reference images. The evaluation used a single scanner to focus on the variation in the staining process. However, not only the staining process needs to be addressed, but also the scanner device dependency. Since the color of the calibrator microbeads is obtained from the scanned image, the scanner characteristics may be corrected during the above calibration process, but this has not been investigated. Moreover, there is another question we should address; would there be any benefit to applying color correction using a CCS for color variations due to differences in scanning devices.

This paper introduces scanner color correction using the CCS into the previous calibration method (Fig. 3), to address the color variations depending on the WSI scanners. It is assumed that the reference and target datasets would be scanned with different WSI scanners (Fig. 3). However, in practical use, the reference and target scanners can either be the same or differ. The reference dataset includes the tissues of reference breast cancer cases, the IHC-CS stained with the same batch as the reference tissues. They are digitized by scanner A together with the CCS. Similarly, the target tissues are also stained simultaneously with the IHC-CS and digitized by scanner B together with CCS.

Based on the colorimetric characterization using the CCS, the pixel value of the scanned image is corrected. After that, the DAB color and intensity calibration using the IHC-CS is applied, as previously reported. There are two options for DAB color and intensity calibration, method1 and method2, as described in the subsection of "Staining color and intensity calibration." Finally, the images are evaluated with existing automated image analysis software for HER2.

### Color calibration using color chart slide

**Fig. 4:**
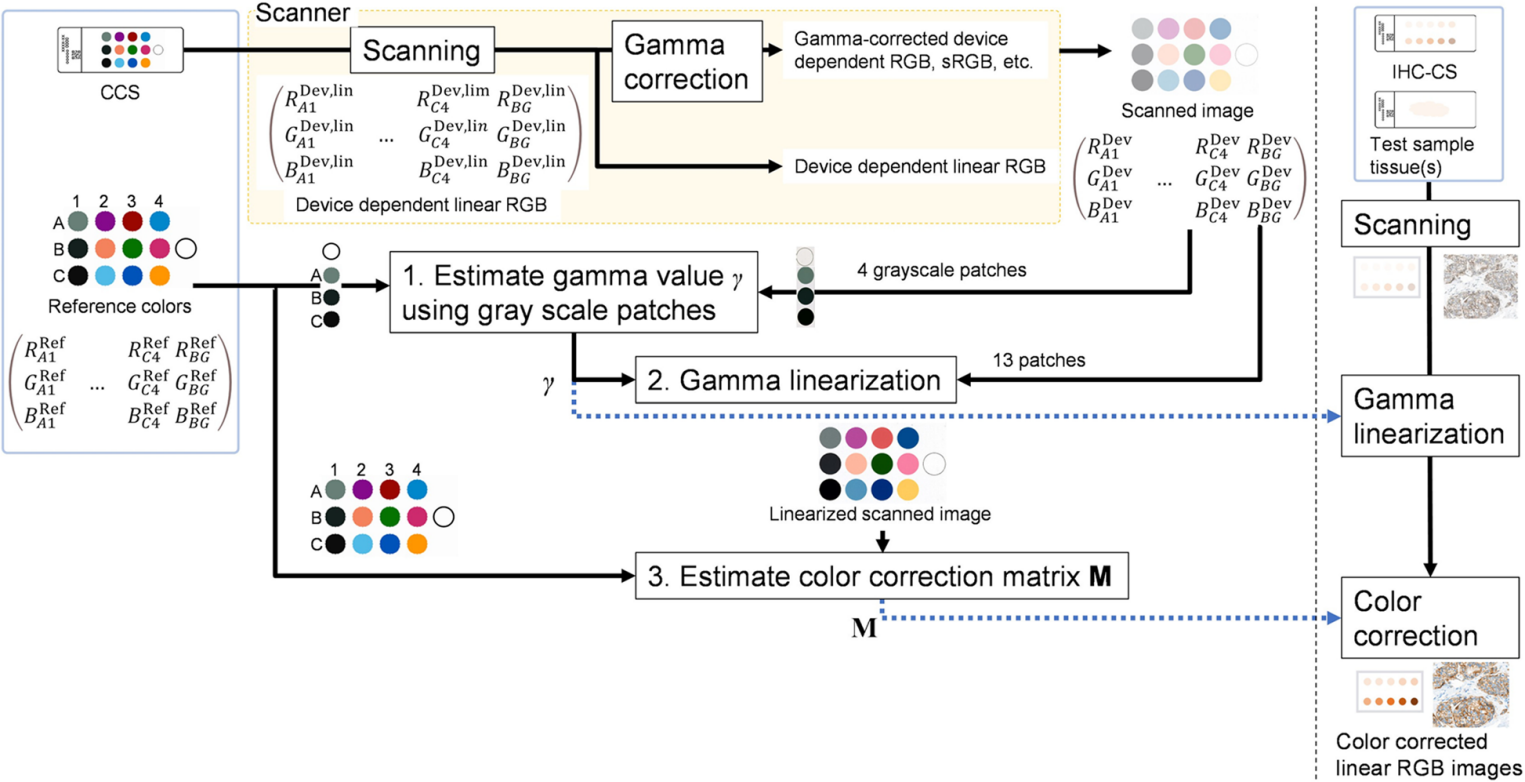
Flow of scanner calibration. Reference and scanned colors of the color chart slide are used to obtain the parameters for calibration. Gamma linearization and color correction are performed on each image to calibrate the scanned color to the reference colors of the color chart slide

Figure 4 shows the process of color calibration for adjusting scanner device characteristics. It is a simplified method for the colorimetric characterization of an input device. Stained slides and the CCS need to be scanned with the same scanner setting. We define a linear RGB color space as a standard device-independent color space for all image analysis processing. The white point is standard illuminant D65, and R, G, and B primary colors defined in sRGB standard are used.

The RGB values output by the scanner may be device-dependent or conform to a standard color space such as sRGB. The characteristics of RGB filters or illumination light depends on scanners, and the same color object often results in different RGB values. In addition, some scanners can produce linear RGB values, but the RGB values in most WSIs are often gamma-corrected, as gamma correction is necessary for image display standards. Gamma $\ne$ 1 means the nonlinear tone curve. Therefore, the device color calibration must address both the matrix-based color space conversion and the gamma linearization.

To derive the parameters for the gamma linearization and matrix-based color correction, reference and scanned colors of the patches in the CCS were used. After obtaining the gamma value for linearization and the color correction matrix for color space conversion, both steps are applied to each scanner image. Finally, a color-corrected linear RGB image in the standard device-independent color space is obtained.

### Reference colors in the standard linear RGB space

The CCS accompanies NIST traceable calibration data of spectral transmittance *T*(*λ*) ranging from 340 to 830 nm at 5 nm intervals. The CIEXYZ tristimulus values in CIE 1931 XYZ color space are calculated using the spectral distribution of the illuminant source and the CIE 1931 color-matching functions. In this research, illuminant D65 was used as the light source *S*(*λ*). Obtained tristimulus values ${\varvec{T}}={\left(X Y Z\right)}^{t}$ are converted to the linear RGB values ${\varvec{R}}={\left(R G B\right)}^{t}$ by multiplying with coefficients of 3 × 3 XYZ to RGB conversion matrix ***C,*** as:

1 $${\varvec{T}}={\varvec{C}}{\varvec{R}} .$$

where *t* denotes the matrix transpose. The reference RGB values of the 13 color patches in the CCS are calculated using Eq. (1), and stored in a 3 × 13 matrix ***G***r, which contains the linear RGB values of 12 color patches and background as follows:

2 $${{\varvec{G}}}_{r}=\left(\begin{array}{ccc}{R}_{A1}^{\mathrm{Ref}}& & {R}_{C4}^{\mathrm{Ref}}\\ {G}_{A1}^{\mathrm{Ref}}& \dots & {G}_{C4}^{\mathrm{Ref}}\\ {B}_{A1}^{\mathrm{Ref}}& & {B}_{C4}^{\mathrm{Ref}}\end{array} \begin{array}{c}{R}_{BG}^{\mathrm{Ref}}\\ {G}_{BG}^{\mathrm{Ref}}\\ {B}_{BG}^{\mathrm{Ref}}\end{array}\right) ,$$

where the subscripts A1, … C4 represent the indices to the color patches shown in Fig. 2 (a), and BG represents the background patch. The superscript Ref indicates the RGB values of the reference. Figure 2 (b) shows the calculated reference colors (in sRGB color space).

### Scanned colors

The color space of the scanned image is either gamma-corrected RGB or linear RGB space, where gamma = 1 in the linear RGB case. The RGB values at each pixel are normalized by dividing with incident light RGB as:

3 $${{\varvec{R}}}^{\mathrm{Dev}}={\varvec{I}}\oslash {{\varvec{I}}}_{0} ,$$

where ${{\varvec{R}}}^{\mathrm{Dev}}= {\left({R}^{\mathrm{Dev}} {G}^{\mathrm{Dev}} {B}^{\mathrm{Dev}}\right)}^{t}$ is the normalized device-dependent RGB vector at each pixel in the image, ***I***_0_ $= {\left({I}_{0\mathrm{R}} {I}_{0\mathrm{G}} {I}_{0\mathrm{B}}\right)}^{t}$ is the RGB intensity vector of the incident light obtained from the glass region of the CCS, ***I*** $= {\left({I}_{\mathrm{R}} {I}_{\mathrm{G}} {I}_{\mathrm{B}}\right)}^{t}$ is the RGB intensity vector of each pixel, and ⊘ denotes Hadamard division operator, respectively.

From the scanned image of the CCS, the average value of the central area of each patch is calculated, then a 3 × 13 matrix **Gs**, which contains the scanned normalized RGB values, was obtained:

4 $${{\varvec{G}}}_{s}=\left(\begin{array}{ccc}{R}_{A1}^{\mathrm{Dev}}& & {R}_{C4}^{\mathrm{Dev}}\\ {G}_{A1}^{\mathrm{Dev}}& \dots & {G}_{C4}^{\mathrm{Dev}}\\ {B}_{A1}^{\mathrm{Dev}}& & {B}_{C4}^{\mathrm{Dev}}\end{array} \begin{array}{c}{R}_{BG}^{\mathrm{Dev}}\\ {G}_{BG}^{\mathrm{Dev}}\\ {B}_{BG}^{\mathrm{Dev}}\end{array}\right) .$$

### Gamma linearization

The linearization process is based on the gamma transformation model formulated by a power law. The standard gamma correction assumes the display gamma, represented by $\gamma$, in which the output light intensity is given by the power $\gamma$ of the input value. If there is a device-dependent linear RGB value, ${D}^{\mathrm{Dev},\mathrm{Lin}}={R}^{\mathrm{Dev},\mathrm{Lin}}, {G}^{\mathrm{Dev},\mathrm{Lin}},\mathrm{or}\ {B}^{\mathrm{Dev},\mathrm{Lin}}$, the gamma-corrected RGB value ${D}^{\mathrm{Dev}}={R}^{\mathrm{Dev}}, {G}^{\mathrm{Dev}},\mathrm{or}\ {B}^{\mathrm{Dev}}$ is given by ${D}^{\mathrm{Dev},\mathrm{Lin}}$ to the ($1/\gamma$) power. Thus, the linearization is performed by

5 $${D}^{\mathrm{Dev},\mathrm{Lin}}={\left({D}^{\mathrm{Dev}}\right)}^{\gamma } ,$$

where $\gamma$ is the parameter required for the linearization process derived from the luminance Y of grayscale color patches (A1, B1, C1, and background in Fig. 2 (b)). The least square method was used to find an optimal $\gamma$ for Eq. (5) that best fits the reference and scanned colors. If the color profile of the scanned image is linear ($\gamma$ =1), scanned values equal the reference values producing a straight line. Otherwise, the obtained $\gamma$ corrects the pixel value of the scanned image.

### Derivation of color correction parameters by regression

Color correction by nonlinear regression (Cheung et al. 2004) is used in this system. ${\varvec{M}}\left\{\right\}$ is a three-dimensional column vector of the nonlinear regression functions for R, G, and B. To determine the order of polynomial transformation, the color difference dE* explained in the subsequent subsection is calculated after color calibration for different combinations of orders of two scanners. Then, the combination with the minimum color difference is selected. The polynomial order used in the regression is experimentally determined to be 5, as in Eq. (6). The regression parameters in ${\varvec{M}}\left\{\right\}$ are derived using the reference and scanned color matrices ***G***r and ***G***s.

6 $$\left(\begin{array}{c}{R}^{\mathrm{Cor}}\\ {G}^{\mathrm{Cor}}\\ {B}^{\mathrm{Cor}}\end{array}\right)={\varvec{M}}\left\{ \begin{array}{c}{M}_{R}\left({R}^{DEV},{G}^{DEV}, {B}^{DEV},{R}^{DEV}{G}^{DEV}{B}^{DEV},1\right)\\ {M}_{G}\left({R}^{DEV},{G}^{DEV}, {B}^{DEV},{R}^{DEV}{G}^{DEV}{B}^{DEV},1\right)\\ {M}_{B}\left({R}^{DEV},{G}^{DEV}, {B}^{DEV},{R}^{DEV}{G}^{DEV} {B}^{DEV},1\right)\end{array}\right\}$$

Another approach for finding ${\varvec{M}}\left\{\right\}$, white-point preserved least-square (WPPLS) has been reported (Finlayson and Drew. 1997a, 1997b). This method finds ${\varvec{M}}\left\{\right\}$ that minimizes the overall residual square error and, at the same time, preserves the background white:

7 $${\varvec{G}}_{\mathrm r}={\varvec{M}}\left\{{\varvec{G}}_S\right\}\;and\; {\varvec{M}}{\{{\varvec{u}}\mathit\}}\;=\;{\varvec{u}},$$

where ***u*** is a column vector of the background white, equal to (1,1,1)^t^. In this study, WPPLS with polynomial transform is used.

### Color difference between images

The color difference between images was assessed using the color difference in the CIE 1976 L*a*b*, or CIELAB color space. The XYZ tristimulus values are transformed to the uniform chromaticity space, L*a*b*, and the Euclidian distance is calculated as follows:

8 $${\mathrm{dE}}^{*}=\sqrt{({\mathrm{L}}_{1}^{*}-{\mathrm{L}}_{2}^{*}{)}^{2}+({\mathrm{a}}_{1}^{*}-{\mathrm{a}}_{2}^{*}{)}^{2}+({\mathrm{b}}_{1}^{*}-{\mathrm{b}}_{2}^{*}{)}^{2}}$$

When dE* is between 1.0 and 2.0, only the experienced observer can notice the perceptual difference, and when dE* is below 1.0, an observer cannot perceive the difference (Mokrzycki and Tatol. 2011).

### Staining color and intensity calibration

**Fig. 5:**
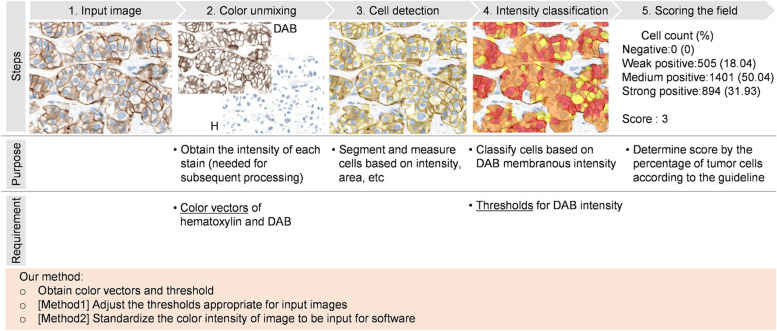
Workflow of HER2 assessment using software and relation between the software and the described method. After color unmixing, cell detection is performed. Average DAB color intensity of each cell membrane is measured and immunoscore is assigned for each cell by comparing of the average DAB color intensity and the thresholds. HER2 status is determined by the percentage of tumor cells in each immunoscore

The use of existing automated image analysis software for the HER2 test is assumed. Figure 5 shows the workflow of HER2 assessment using the standard automated image analysis software. The details of the algorithm may depend on the type of software:

- 1. A color-unmixing method is applied to separate DAB and hematoxylin signals as intensity images.
- 2. Cell detection is deployed on the separated intensity images. Nucleus regions are detected on the hematoxylin intensity image, and membrane regions are detected on the DAB intensity image based on the detected nucleus locations. Intermediate regions between the nuclei and the membrane are classified as the cytoplasm.
- 3. The average DAB intensity of each cell membrane is classified into four groups according to user-set intensity thresholds. Each group is labeled with an immunoscore *m* of 0, 1, 2, or 3, corresponding to no, weak, moderate, or strong staining.
- 4. HER2 status is determined from the percentage of tumor cells classified to each immunoscore according to the ASCO/CAP guidelines.

Depending on the staining process, the color vectors for color unmixing and the stain intensity may vary. Thus, two implementations (method1 and method2) were previously proposed to calibrate the stain color vectors and the DAB intensity for the automated assessment using the IHC-CS (Ohnishi et al. 2022, 2023). Method1 adjusts thresholds appropriate for the input image in intensity classification. Method2 corrects the color intensity of the images to be input for software. Users can select a suitable correction method depending on the automated image analysis software; if the image analysis software is open-source or threshold adjustable, proposed method1 can be applied. Otherwise, proposed method2 will be suitable.

Figure 3 also summarizes the protocol for staining calibration using the IHC-CSs. The protocol consists of two stages: preparation and evaluation. The IHC-CS is stained in both stages to obtain the DAB color intensity for calibrating the various staining conditions.

In the preparation stage, IHC-CS and the HER2 score known breast cancer cases are prepared as a reference dataset. IHC-CS is originally designed for the QA/QC of staining and cannot be used to directly determine HER2 score. Therefore, the score known breast cancer cases are required to obtain the intensity thresholds for classification. After staining all slides together, the slides are digitized by a reference scanner (Scanner A in Fig. 3). The reference stain intensity is calculated from the microbeads of each level in the IHC-CS image, and intensity characteristics are derived from the obtained reference stain intensities. The intensity thresholds are automatically determined from the reference DAB-stained tissue data.

In the evaluation stage, another IHC-CS is stained and scanned with the clinical tissue slides to be assessed (target dataset). The color and stain intensity are estimated from the IHC-CS image and used in the following procedures. In method1, the color unmixing matrix and the appropriate thresholds for score classification are determined from the obtained color and stain intensity. In method2, the color and intensity of the tissue images are corrected by the obtained color and stain intensity. The same thresholds obtained in the preparation stage are commonly used for the color-corrected images. If Scanner A, the same as the preparation stage, is used for the target datasets, proposed protocol implies calibrating the staining condition. If a different scanner from the preparation stage is used, this protocol calibrates both the scanner and the staining condition. The details of this protocol using IHC-CS were presented in (Ohnishi et al. 2022, 2023).

### Evaluation of proposed protocol

The experimental evaluation addresses the following two questions;

- 1) Is the color calibration using the CCS effective in IHC stained tissue and microbeads?
- 2) In the automated evaluation of HER2, can we achieve consistent results when different scanners are used, and color variation is caused by the staining process?

We use Scanner A for the reference scanner in the preparation stage and Scanners A and B for target data in the evaluation stage.

### Color calibration using the CCS

The effectiveness of scanner calibration was evaluated using the CCS for IHC slides to compare with H&E-stained specimens. A slide was scanned with the two scanners and the color difference dE* between the two images was calculated pixel-wise, which generated a dE* map. Each pixel of the dE* map shows the dE* of the same pixel in the two images. Since the images obtained from the two scanners have different spatial dimensions and pixel resolutions, image registration and magnification correction are needed. In this experiment, Accelerated-KAZE (Alcantarilla et al. 2013), which detects and matches key points on two images, was employed for image registration. For the IHC-CS images, a mask image focusing only on the microbead area was manually created to evaluate the dE*. The mean and standard deviation (*SD*) of dE* in each image were calculated. A histogram of each dE* map was created with bin = 64.

### The effect of the proposed calibration in automated HER2 classification

**Fig. 6:**
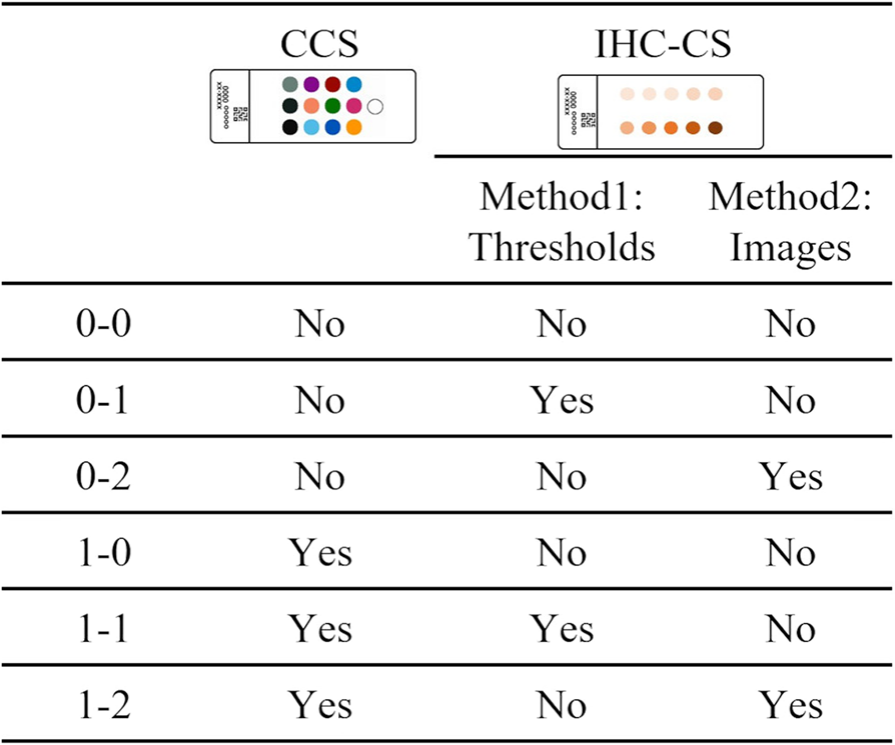
Combinations of the calibration methods

One of the six datasets was devoted as a reference dataset, and the remaining five datasets were the target for the HER2 test. The experiment assumed the target images would be digitalized with various scanner settings containing gamma correction. Therefore, the target datasets were gamma-corrected with a gamma value of 1.8 or 2.2 by randomly selecting datasets. A total of 10 datasets were prepared (Table 1). Figure 6 shows different combinations of the scanner and staining calibration methods. For these six methods, the automatic HER2 evaluation was performed, and concordance with the pathologist’s assessment was compared.

**Table 1:** Combinations of the scanner and gamma value for target datasets

Scanner	Gamma	Number of data
A	1.0 (linear)	2
A	1.8	2
A	2.2	1
B	1.0 (linear)	2
B	1.8	1
B	2.2	2
Total		10

Open-source software QuPath (Bankhead et al. 2017) was used as the image analysis software for HER2 evaluation. This semi-automatic software allows a user to modify the color vectors for color unmixing and the thresholds for classification. Histoscore (H-score), which is one of the evaluation indexes in HER2 assessment, was calculated by:

9 $$\mathrm{H}-\mathrm{score}=\sum \left({P}_{m}\times m\right),$$

where *P* is the percentage of immunoreactive tumor cells, *m* is immunoscore (0, 1, 2, or 3).

Since the target datasets were obtained from the serial sections of the same case, the difference in H-score would be significantly slight if there is no staining and scanning variation. The *SD* of the H-score was calculated to check if the proposed protocol effectively reduced variability in score classification. Two-way within-subject analysis of variance (ANOVA) was employed on the difference in H-score between the target and reference datasets of each HER2 case to see if there was a significant difference between the six methods. Also, Tukey’s honest significant difference (HSD) test was utilized as a post hoc analysis. A *P*-value less than 0.05 was considered statistically significant for both ANOVA and Tukey’s HSD test.

## Results

### Color calibration

**Fig. 7:**
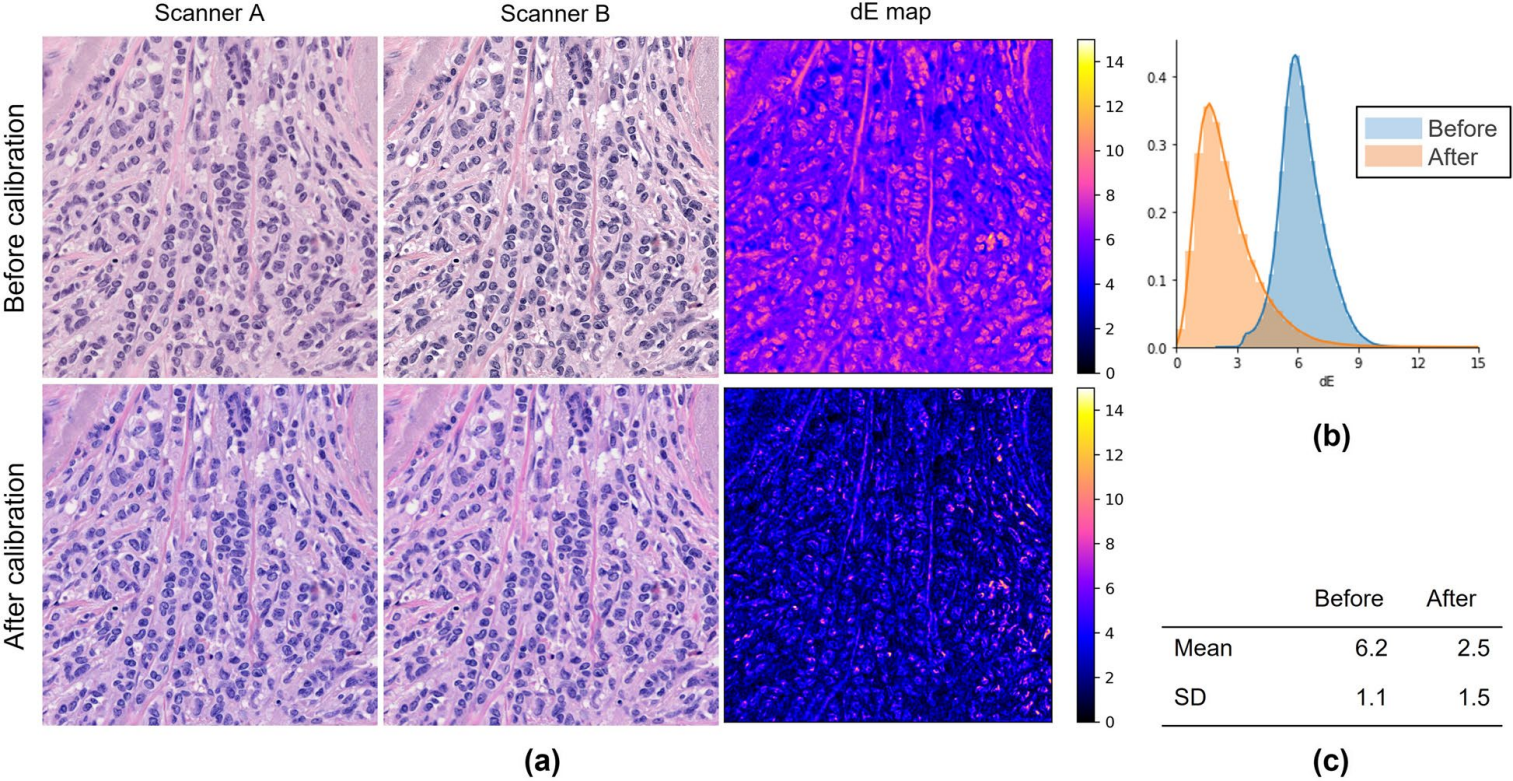
Color calibration on H&E images of breast cancer tissue. **a** Comparison of images before (upper row) and after (lower row) color calibration and dE* maps. The dE* maps show the color differences between two scanners calculated for each corresponding pixel as pixel values; **b** Histograms of the dE* maps; **c** Mean and SD of the dE*

**Fig. 8:**
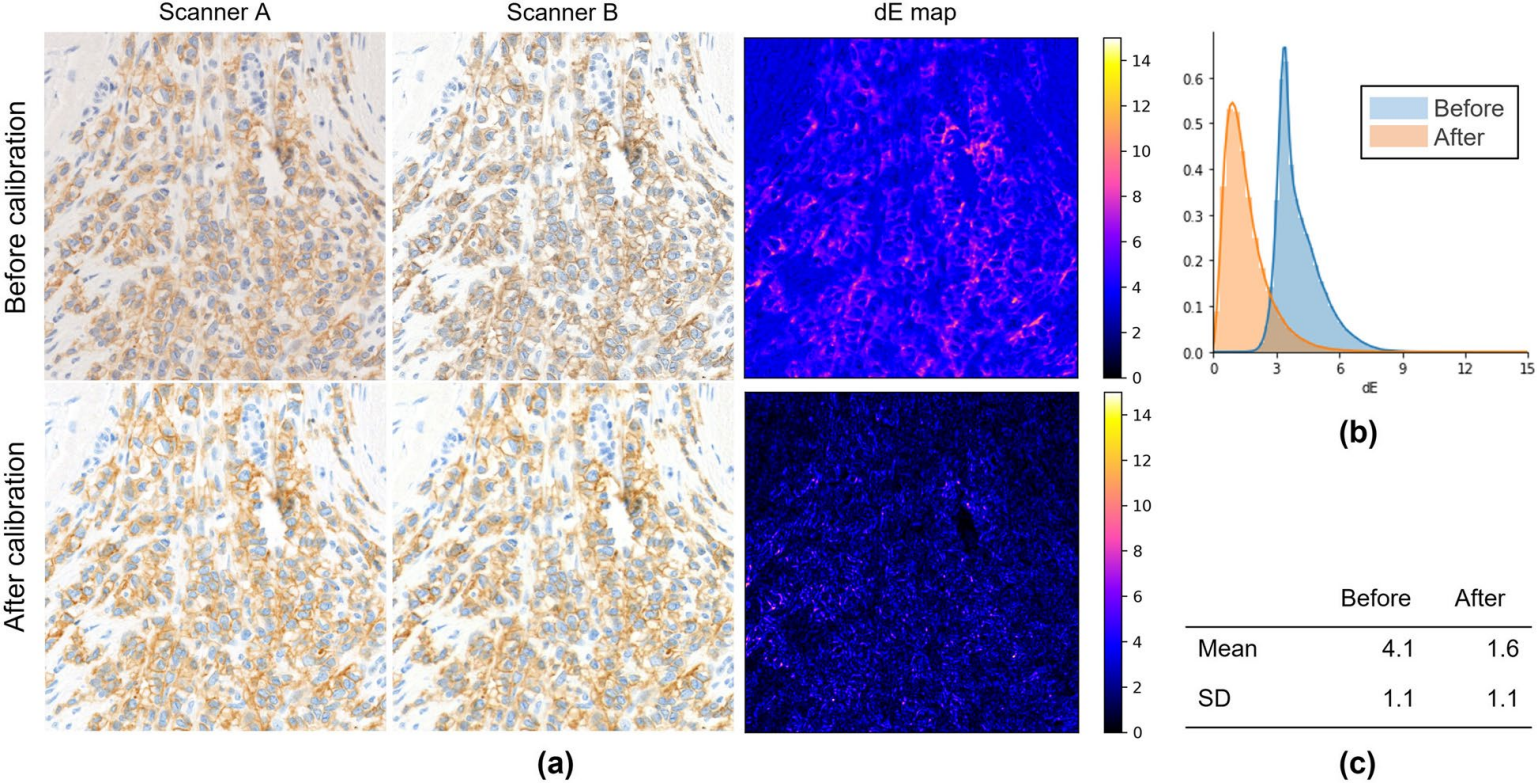
Color calibration on HER2 IHC images of breast cancer tissue scored 2 + . **a** Comparison of images (upper row) and after (lower row) color calibration and dE* maps; **b** Histograms of the dE* maps; **c** Mean and SD of the dE*

**Fig. 9:**
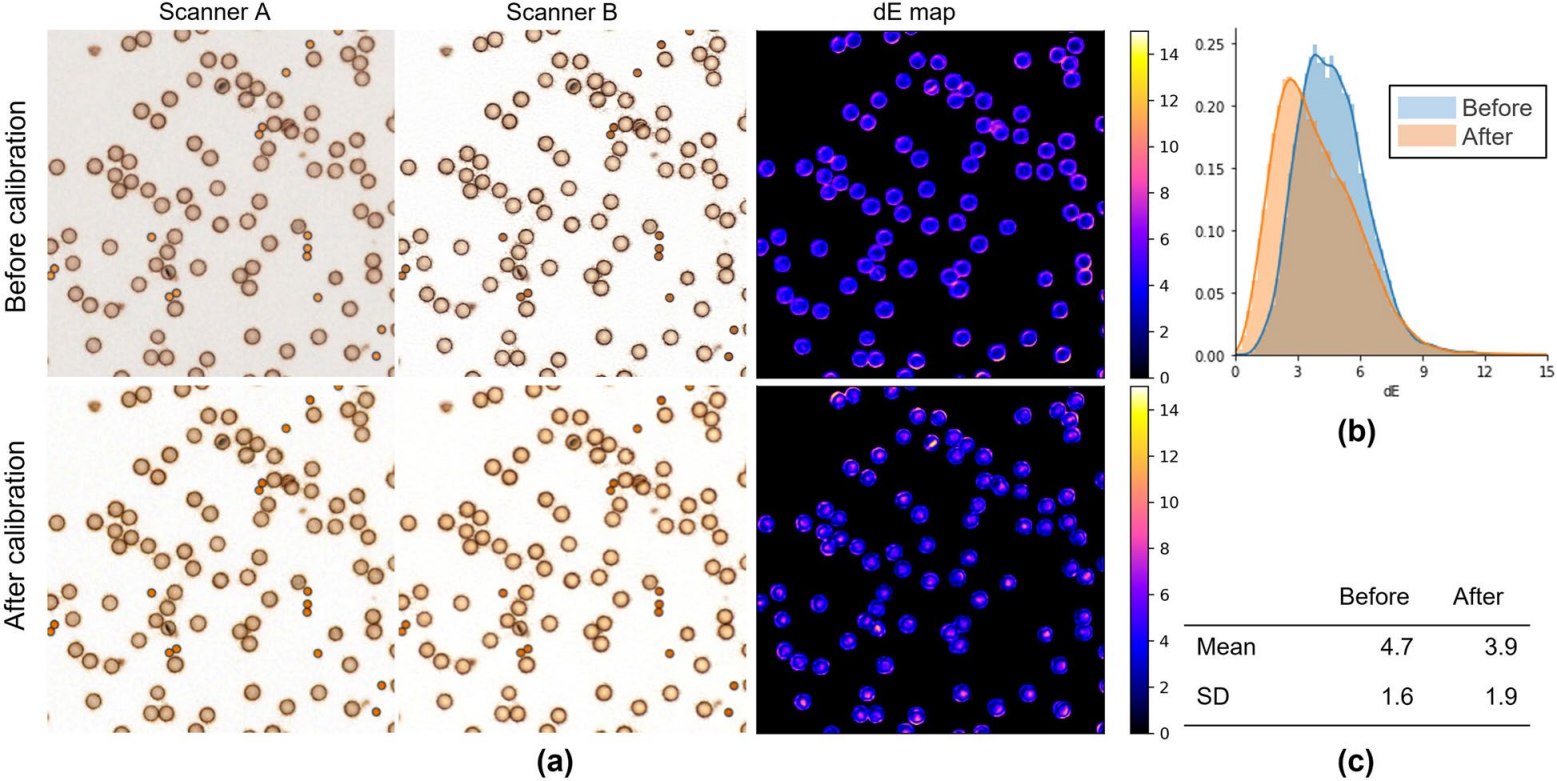
Color calibration for IHC-CS images (level 10). **a** Comparison of images (upper row) and after (lower row) color calibration and dE* maps; **b** Histogram of the dE* maps except for background; **c** Mean and SD of the dE*

Figures 7, 8, 9 show the results of scanner calibration with the CCS. The upper row of Figs. 7, 8, 9 (a) shows uncalibrated images, and the lower row shows the color‑calibrated images. By performing calibration, the mean values of color difference were decreased from 6.2 to 2.5 in H&E stained tissue images, 4.1 to 1.6 in IHC stained tissue images, and 4.7 to 3.9 in IHC-CS images, respectively. By visual assessment of the dE* maps of the H&E stained image, the reduction of dE* in most pixels can be confirmed. In the case of the HER2 IHC images, dE* became lower than 4 in most pixels after calibration with the tissue image. The effect of the calibration was small for the IHC-CS image.

### Automated HER2 assessment

Table 2 shows the result of the automated HER2 assessments. In the results of the uncalibrated procedure (0–0), there were 14 discordance slides out of 30. The discordance was reduced to three slides calibrated with IHC-CS (0–1) and one slide calibrated with CCS (1–0). Applying the calibration with both IHC-CS and CCS (1–1 and 1–2) resulted in a concordance of 100% with the pathologist’s assessment. In addition, the *SD* of the H-score became smaller than uncalibrated procedure with either calibration slide.

**Table 2:** Result of HER2 score classification

	CCS	IHC-CS		Concordance (%)			*SD* of H-score		
Methods		Method1	Method2	1 +	2 +	3 +	1 +	2 +	3 +
0–0	No	No	No	90	30	40	30.3	27.5	47.6
0–1	No	Yes	No	100	100	70	11.0	11.9	27.1
0–2	No	No	Yes	100	100	100	13.3	10.1	16.2
1–0	Yes	No	No	90	100	100	11.2	10.3	8.7
1–1	Yes	Yes	No	100	100	100	7.9	8.4	5.9
1–2	Yes	No	Yes	100	100	100	10.4	7.0	5.3

**Fig. 10:**
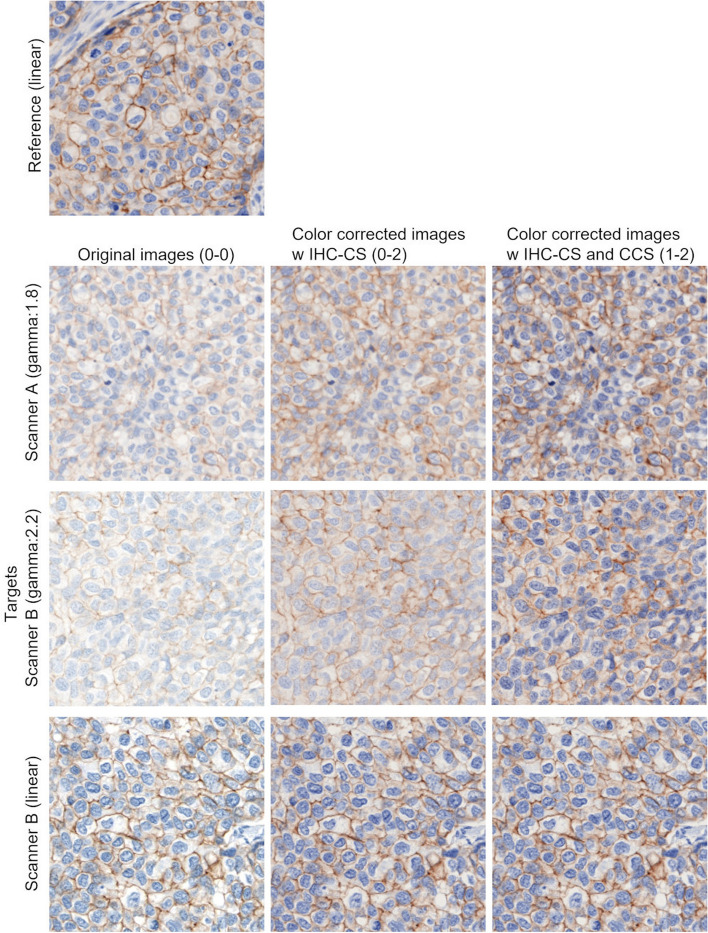
Comparison of original images and color and intensity calibrated images for 2 + case

Figure 10 shows examples of color and intensity calibrated images (0–0, 0–2, and 1–2 in Table 1). When calibrating with IHC-CS only (0–2), only the color and intensity of DAB are subject to calibration. Therefore, for gamma-corrected images, the color and intensity of hematoxylin were not calibrated, and the calibrated images still looked bright. For linear RGB images, color and intensity can be approached to the reference image by calibrating DAB. Gamma linearization was performed when calibrating with CCS and IHC-CS (1–2), and both DAB and hematoxylin could be calibrated.

**Fig. 11:**
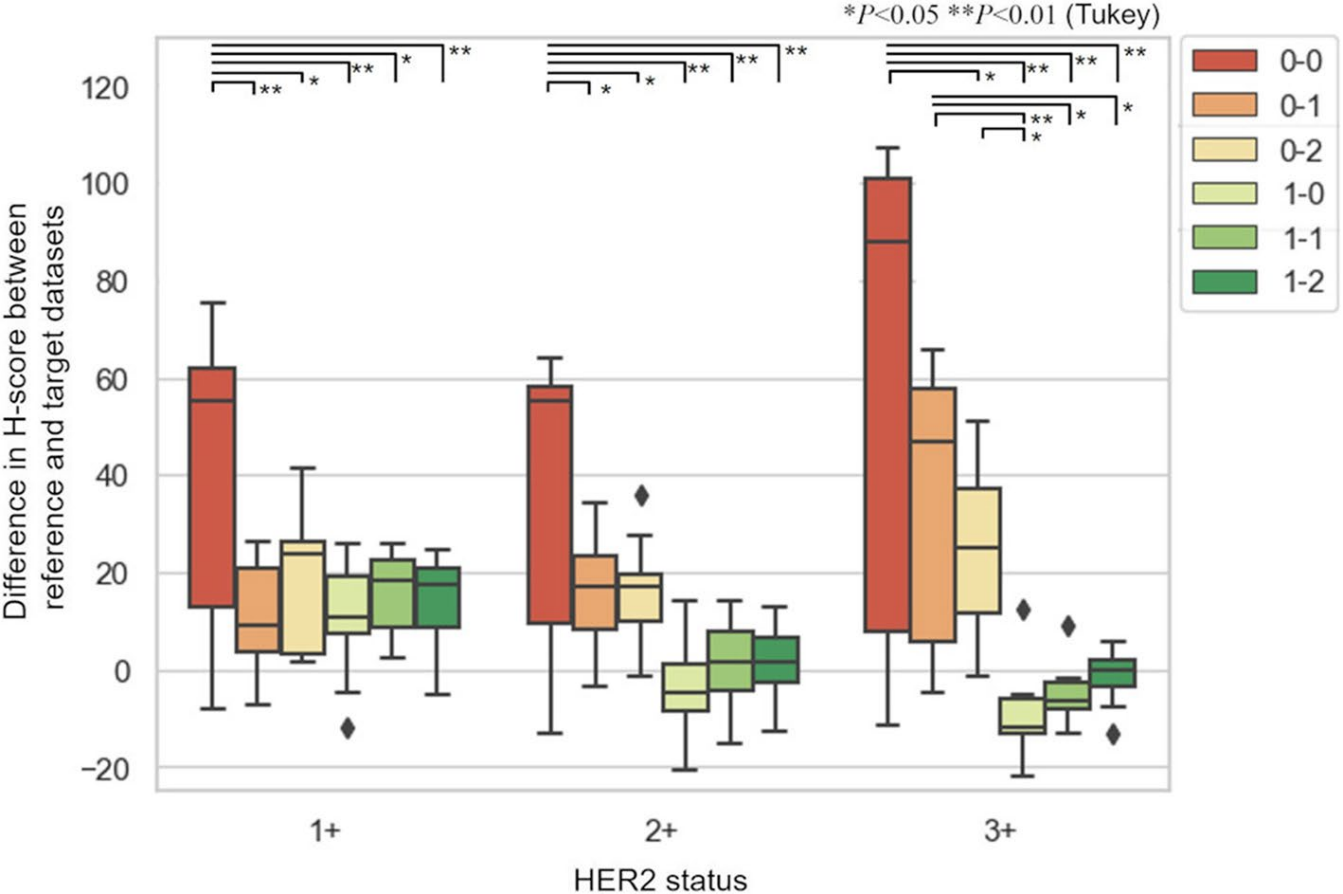
Box plots of the difference in H-score between target and reference image. Vertical bars, central rectangles and horizontal lines within the rectangles represent the minimum to maximum range, interquartile range and median value, respectively. * represents the significant differences by Tukey’s HSD test

Figure 11 shows the box plots of the difference in H-score between the target and reference datasets of each HER2 case. A two-way ANOVA revealed that there were statistically significant interactions between the effect of the scanner and staining calibrations for 1 + case, *F*(2,54) = 5.70, *P* = 0.006, for 2 + cases, *F*(2,54) = 4.53, *P* = 0.015, and for 3 + case, *F*(2,54) = 3.69, *P* = 0.031. Table 3 shows the results of Tukey’s HSD test in cases with significant differences.

**Table 3:** Result of Tukey’s HSD test

HER2 score					95% C.I	
	group1	group2	Mean diff	*P*	Lower	Upper
1 +	0–0	0–1	-28.53 ^**^	0.003	-54.20	-2.87
	0–0	0–2	-21.82 ^*^	0.043	-43.19	-0.45
	0–0	1–0	-29.25 ^**^	0.002	-54.92	-3.59
	0–0	1–1	-24.45 ^*^	0.016	-45.82	-3.08
	0–0	1–2	-26.62 ^**^	0.007	-52.29	-0.95
2 +	0–0	0–1	-20.91 ^*^	0.034	-40.81	-1.01
	0–0	0–2	-20.63 ^*^	0.038	-40.53	-0.73
	0–0	1–0	-39.44 ^**^	0.001	-63.34	-15.53
	0–0	1–1	-35.43 ^**^	0.001	-59.33	-11.52
	0–0	1–2	-35.33 ^**^	0.001	-59.24	-11.43
3 +	0–0	0–2	-34.31 ^*^	0.039	-67.46	-1.16
	0–0	1–0	-68.91 ^**^	0.001	-108.73	-29.10
	0–0	1–1	-64.86 ^**^	0.001	-104.67	-25.04
	0–0	1–2	-61.15 ^**^	0.001	-100.96	-21.34
	0–1	1–0	-43.75 ^**^	0.004	-83.56	-3.93
	0–1	1–1	-39.69 ^*^	0.010	-72.84	-6.54
	0–1	1–2	-35.99 ^*^	0.026	-69.14	-2.84
	0–2	1–0	-34.60 ^*^	0.036	-67.75	-1.45

^*^P < 0.05

^**^P < 0.01

## Discussion

### Scanner color calibration using color chart slide

**Fig. 12:**
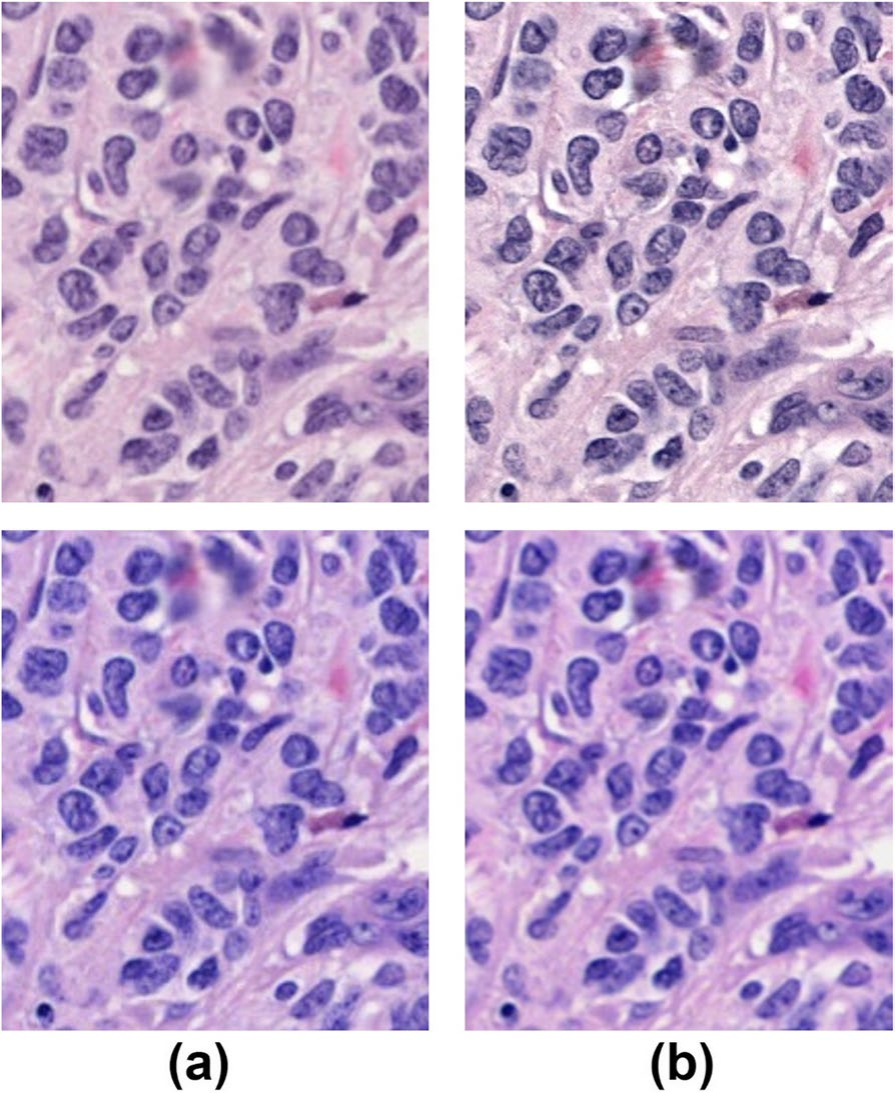
Magnified figure of H&E stained tissue images. **a** and **b** Scanner A and B before (upper) and after (lower) calibration

**Fig. 13:**
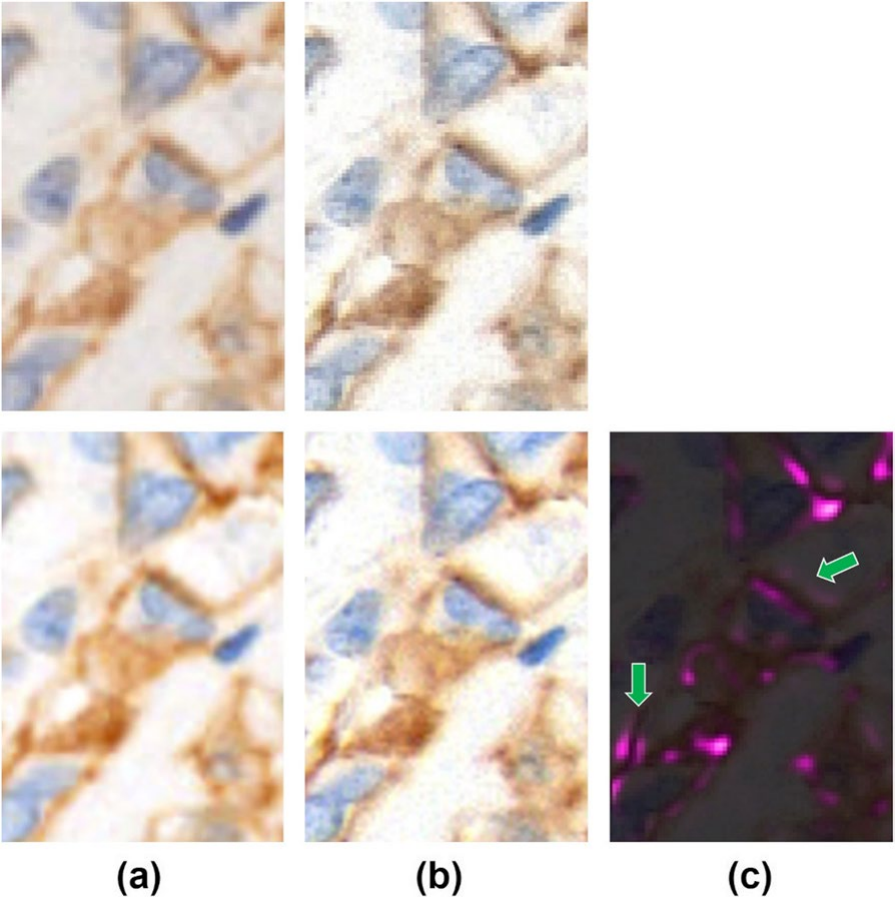
Comparison of tissue images. **a** and **b** Scanner A and B before (upper) and after (lower) calibration; **c** Superimposed image of dE* map in magenta and scanner B. Green arrows indicate that dE* is large due to the difference of spatial frequencies between scanners

The proposed protocol utilizing the CCS allows one to standardize the color of WSI, which varies depending on scanning devices. The color differences between scanners have been decreased in both H&E and IHC stained specimens using this protocol. In H&E, the change in the nuclei is noticeable (Fig. 12). In IHC, the mean dE* was decreased to less than 2.0, which means only an expert observer can notice the difference. It should be noted that when comparing images scanned by different scanners, the differences in scanner configuration, such as image resolution, numerical aperture, and so on need to be considered. When creating the dE* map, the images scanned by Scanner B were downscaled to match the resolution to those of Scanner A. However, for the visual impression, the sharpness and contrast of the images from Scanner B were better even after downscaling (Fig. 13 (a)(b)). Therefore, larger color differences remained at brighter and darker pixel values as indicated by green arrows in Fig. 13 because the scanner calibration with CCS could not be satisfactorily corrected. The histograms after calibration in Figs. 7, 8, 9 (b) have a tail on the right side, which may be related to the color difference caused by factors that cannot be calibrated with CCS.

Scanner calibration decreased the color difference in the calibrator image as well. However, the effect was not as good as in the tissue images. This may be a scanning issue. IHC-CS is comprised of two different sizes of microbeads. When the smaller microbeads are in focus, the larger microbeads appear brighter in the image. This changes in brightness caused by focus may not be calibrated with the CCS. In this experiment, when scanning the IHC-CS with Scanner B, the focus range was set so that the larger microbeads were in focus. This process would be a limitation for clinical use. The IHC-CS is used for a different purpose than originally intended in the commercialized product. We are considering improving the calibrator.

### HER2 assessment with color chart slide and IHC-calibrator slide

The effectiveness of different combinations of the scanner and staining calibrations was evaluated by the concordance between manual and automated HER2 assessment. In the results of the uncalibrated procedure (0–0), there were 14 discordance slides out of 30. Two discordance slides out of 14 were caused by staining variation because the slides were digitalized with the same scanner and settings as the reference. In the remaining 12 slides, the discordances were due to the scanner and staining variations. The target datasets included linear and gamma-corrected RGB images, and discordances were observed, especially for gamma-corrected images. In the gamma-corrected image, pixel values of the dark area became lighter. Since the linear RGB images were used as a reference, evaluating the gamma-corrected images with the thresholds appropriate for linear RGB reference would result in the strong staining being evaluated as weak staining. Applying the calibrations with IHC-CS and CCS (1–1 and 1–2) resulted in a concordance of 100% with the pathologist’s assessment. *SD* of H-score became smaller in most cases by applying calibrations. For the 1 + case, the evaluated invasive region was small compared with other cases, and H-score was likely to change significantly with the changes in the classification of the cell immunoscore. All IHC slides were stained with the FDA-approved antibody and slide stainer, so the variation in staining was inherently smaller than the variation in scanning. Therefore, the difference between method (1–0) and methods (1–1) and (1–2) was small.

**Fig. 14:**
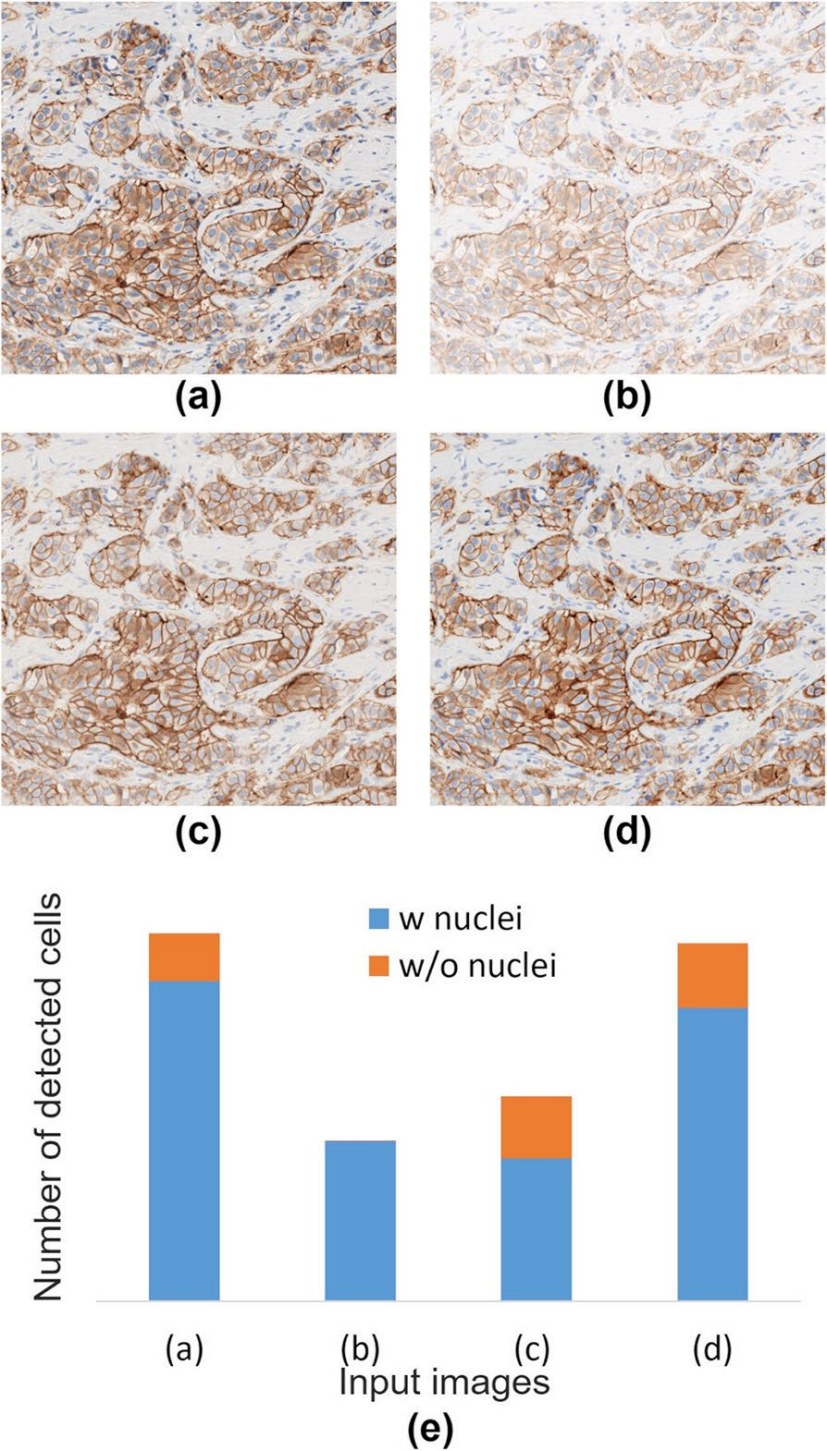
Differences in cell detection results by software in 3 + case. **a** Linear RGB image; **b** – **d** gamma corrected images, uncalibrated, calibrated with IHC-calibrator slide (method:0–2), and calibrated with color chart slide and IHC-calibrator slide (method:1–2); **e** Comparison of number of cells detected by the software for each input image. Blue: cells with nuclei detected, and orange: cells without nuclei detected

**Fig. 15:**
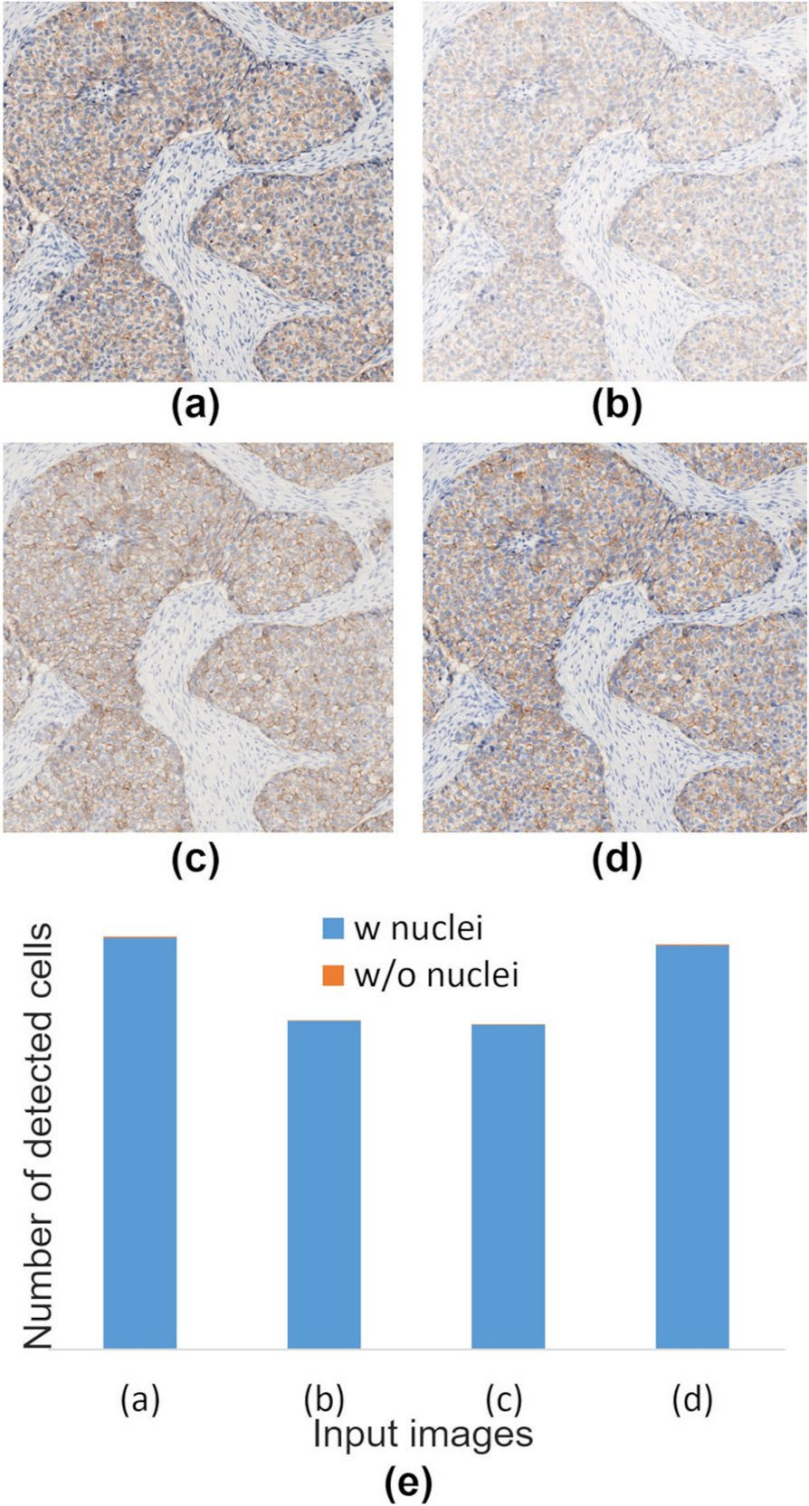
Differences in cell detection results by software in 2 + case. **a** Linear RGB image; **b** – **d** gamma corrected images, uncalibrated, calibrated with IHC-calibrator slide (method:0–2), and calibrated with color chart slide and IHC-calibrator slide (method:1–2); **e** Comparison of number of cells detected by the software for each input image. Blue: cells with nuclei detected, and orange: cells without nuclei detected

For the 3 + case, the two methods using IHC-CS (0–1 and 0–2) produced different results. This difference may be due to the algorithm of the analysis software. The relationship between the input images and the results of cell detection by the analysis software was examined using 3 + tissue images (Fig. 14). The four images were (a) linear RGB image, (b)-(d) gamma-corrected image with a gamma value of 2.2, uncalibrated, calibrated with IHC-CS, and calibrated with CCS and IHC-CS. Each image was input into the software and analyzed with the same settings. The number of detected cells was compared (Fig. 14 (e)). The software used in this study detects cells from the intensity images of respective hematoxylin and DAB. Some cells have only membranes and no nuclei detected, but these cells are also used for HER2 evaluation. The number of cells detected by analyzing gamma-corrected image was less than half the number of cells detected by analyzing linear RGB image, perhaps because the unmixed hematoxylin and DAB intensities were weak for cell detection. By calibrating the DAB intensities with IHC-CS, the total number of detected cells increased because the number of cells detected from DAB intensities image increased. However, since intensities of hematoxylin were not calibrated, the number of detected cells is still about half that of the linear RGB image. By using CCS and IHC-CS, the number of detected cells was close to the linear RGB image. A similar result was observed for the 2 + case (Fig. 15). The results of score classification of 2 + case were correct for methods (0–1) and (0–2), but reliability of the results of method (0–1) was less than those of method (0–2) (Table 2).

The difference in results by method (0–1) between 3 + case and other cases may be due to the characteristics of DAB. It has been pointed out that darkly stained DAB violates Lambert Beer’s law due to scattering caused by DAB (Van der Loos. 2008). For the 3 + case, in regions with high DAB intensities, a small amount of DAB component may be separated as hematoxylin component in color unmixing. With gamma-corrected images, even if the intensities of these false regions are low, it is difficult to detect cells from nuclei image because the intensities of nuclei regions are also low. With linear RGB images, the effect for cell detection is small because false regions’ intensities are lower than actual nuclei regions. In general, it also causes difficulty in estimating the DAB stain vector but does not impact the proposed method because it is estimated from the lightly stained microbeads. Moreover, the thresholds used in the proposed method ranged from 0.03 to 0.29 optical density units. Thus, the error in the darkly stained region does not seem to affect the classifications. It is desirable to upgrade the color unmixing model and the algorithm for quantifying the HER2 score in the future.

### Introduction of calibration slides for clinical use

There already exists FDA-approved software for automated HER2 score assessment. However, it is often a package of systems, including the slide stainer, WSI scanner, and software. Introducing such systems requires adapting a new staining workflow or an intended WSI scanner and dramatic modification of the clinical workflow. The modification is not realistic in the clinical setting, and it is a substantial disincentive to introduce an automated image analysis system. The proposed protocol can easily be introduced since the required additional process for adapting the proposed protocol is only using the IHC-CS and CCS in the existing staining and scanning workflows. The described methos can be applied even in FDA-approved systems. When using packaged FDA-approved software, a scanner endorsed by FDA should be used as the reference scanner. By applying the proposed protocol with another scanner as a target scanner, the images digitalized by such scanner could be analyzed by FDA-approved software. It is expected to conduct demonstrations in a larger scale and more realistic environment on the next step.

## Conclusions

In this study, we proposed a protocol for the standardization of staining and scanner variations for the automated IHC assessment. First, the effect of the CCS was evaluated. Color differences between scanners were decreased with calibration for both H&E and IHC stained tissue images. Also, a comparison of the automated HER2 evaluation results was performed using the IHC calibrator alone and a combination of the CCS and the IHC-CS. From the experimental results on breast cancer cases, we confirm that the automated analysis with both scanner and staining calibration showed concordance of 100% with the pathologist’s assessment. When linear RGB images were targeted, IHC-CS could calibrate the color and intensity variation caused by the staining and scanning device. When targeting gamma-corrected images, it is preferable to calibrate using CCS. In practice use, it is expected that gamma-corrected images will also be subject to evaluation. It is recommended to perform calibration using CCS and IHC-CS.

## Abbreviations

HER2: Human epidermal growth factor receptor 2
IHC: Immunohistochemistry
WSI: Whole Slide Image
IHC-CS: IHC-calibrator slide
CCS: Color chart slide
FDA: Food and Drug Administration
DAB: 3,3′-Diaminobenzidine
ASCO/CAP: American Society of Clinical Oncology /College of American Pathologists
H-score: Histoscore
SD: Standard deviation
ANOVA: Analysis of variance
Tukey’s HSD test: Tukey’s honest significant difference test
